# Fostering Spiritually Supportive Work Environments in Healthcare: Leadership Recommendations From a Narrative Review

**DOI:** 10.1155/jonm/4082021

**Published:** 2026-04-21

**Authors:** Rachel A. Joseph, Ashley Hudson Tharpe, Holly Ortman, J. Michelle Crager

**Affiliations:** ^1^ Liberty University, Lynchburg, Virginia, USA, liberty.edu

**Keywords:** nursing, religiosity, spirituality, spiritually supportive environment, well-being

## Abstract

**Background:**

Spiritual support creates a positive clinical work environment for nurses. The literature increasingly shows that spiritually supportive settings improve nurse well‐being, reduce burnout, and elevate patient care quality.

**Aim:**

This narrative review examines how different elements shape spiritually supportive work environments and support nurse well‐being, drawing on peer‐reviewed publications from 2020 to 2025 and data from major national professional organizations.

**Methods:**

A comprehensive literature review was conducted using peer‐reviewed publications from 2020 and 2025, along with data from major national professional organizations. Findings were analyzed to examine how different elements shape spiritually supportive work environments and support nurse well‐being.

**Results:**

The analysis reveals that spiritually supportive settings significantly improve nurse well‐being, decrease burnout, and elevate patient care quality. These environments also yield organizational benefits, such as higher nurse retention and enhanced patient satisfaction. The literature highlights that spirituality and religiosity foster resilience, reduce moral distress, and strengthen nurse–patient relationships, contributing to a compassionate workforce.

**Conclusion:**

Recommendations are to embed spiritual support in healthcare settings to mitigate nurse burnout and workforce shortages, ensuring high‐quality, patient‐centered care. Additional recommendations are to advocate for systemic changes, including spiritual leadership and inclusive policies, to cultivate environments that nurture both nurses and patients, positioning spiritual support as a cornerstone of quality, sustainable healthcare delivery.

## 1. Introduction

In recent years, the integration of spirituality into healthcare institutions has garnered increasing attention from practitioners, universities, and researchers. This growing interest stems from a recognition that spirituality, often defined as a sense of connection to something greater than oneself, can play a pivotal role in the holistic well‐being of patients and all stakeholders. As healthcare systems strive to provide comprehensive care, addressing the spiritual needs of patients has become an essential component of patient‐centered care. The American Association of Colleges of Nursing (AACN), in its 2021 Essentials, emphasized holistic care of patients that includes spiritual care and has endorsed a resolution calling for sustained, systemic action to promote nurse well‐being. This resolution emphasizes the importance of supportive workplace environments, access to mental health resources, and institutional policies that address the holistic needs of nurses [1]. Besides, the American Association of Critical‐Care Nurses (AACN) had developed standards for establishing and sustaining healthy work environments (HWEs) as early as 2005 and revised them in 2016 to describe the optimal work environment that promotes nurse wellness and supports patient outcomes [2]. The Joint Commission [3] requires accredited organizations to assess patients’ spiritual needs, beliefs, values, and preferences of patients and provide supportive care. Additionally, the American Nurses Association [4] in the Code of Ethics in Provision 1.2, 1,3, and 5.2 acknowledges the need for spiritual care for patients.

Spirituality in healthcare is not confined to religion, but it encompasses a broader spectrum of beliefs and values that contribute to an individual’s sense of purpose and meaning. It is important to distinguish spirituality from religiosity. Religiosity refers to organized participation in religious rituals, doctrines, and faith communities, whereas spirituality is a broader construct that includes an individual’s search for meaning, purpose, connectedness, and transcendence, which may or may not involve religious belief [5, 6]. Spirituality may also encompass existential dimensions such as hope, forgiveness, inner peace, and a sense of coherence in the face of suffering [7]. This distinction is important for healthcare organizations seeking to support a diverse workforce, as spiritual support strategies must be inclusive of both religious and nonreligious expressions of meaning‐making. In the context of nurse wellness, spiritual well‐being refers to the degree to which nurses experience meaning, purpose, and emotional wholeness in their professional and personal lives, and this well‐being is shaped by both individual practices and organizational conditions [8, 9]. In this review, spiritually supportive environments are conceptualized as a critical dimension of HWEs, extending existing frameworks by emphasizing meaning, purpose, and connectedness as integral components of workforce well‐being. Research has shown that spiritual well‐being can significantly impact physical and mental health, and overall quality of life [10, 11]. For instance, patients who engage in spiritual practices often exhibit lower levels of stress, improved coping mechanisms, and enhanced recovery rates [12, 13]. Despite the evident benefits, the integration of spirituality into healthcare settings presents several challenges. These include the need for appropriate training for healthcare providers, the development of standardized protocols, and the establishment of a supportive environment that respects diverse spiritual beliefs. Addressing these challenges requires a collaborative effort among healthcare professionals, spiritual care providers, and policymakers. This manuscript aims to explore the various dimensions of spirituality in healthcare, highlighting its importance, the challenges faced, and potential strategies for effective integration. By examining emerging trends and research, we seek to provide a comprehensive narrative review that can inform and guide healthcare institutions as they strive to facilitate holistic patient care. Narrative reviews allow researchers to describe what is known on a topic and are often used for topics that require a meaningful synthesis of complex and broad interpretation [14].

There remains a lack of clarity regarding what constitutes “spiritual support” within fast‐paced clinical environments. Although spiritual well‐being is recognized as an important factor in reducing burnout, compassion fatigue, and emotional strain among nurses, leaders often receive broad or inconsistent guidance that does not translate into practical action steps [15–17]. Literature provides few nonreligious, measurable definitions that organizations can use to support a diverse workforce, leaving leaders without clear tools for implementation. A second gap relates to the persistent divide between recommendations offered in the literature and the structural conditions within healthcare organizations. While transformational leadership frameworks are frequently encouraged, current research offers limited evidence‐based strategies for addressing systemic contributors to distress, such as inadequate staffing, high workloads, and ethically challenging practice environments [8, 18, 19]. When wellness initiatives focus primarily on resilience or self‐care at the individual level, organizational challenges remain unaddressed, which increases the risk of moral distress and moral injury as clinicians find themselves unable to act in alignment with their professional values due to organizational constraints [20–22].

Currently, leaders lack clear guidance on how to prevent or mitigate these issues at a system level. These gaps highlight the need for clearer definitions, practical tools, and organizational frameworks that can guide leaders in supporting the spiritual health and well‐being of the workforce in meaningful and sustainable ways. Current research on holistic care highlights persistent challenges related to defining spirituality and implementing supportive structures within healthcare environments. Although the existing literature primarily focuses on spirituality’s effects on patient outcomes and end‐of‐life care, far fewer studies examine how spiritual support influences workforce health, leadership practices, or organizational climate. Additionally, the current research does not offer a unified framework that links spiritual support to the AACN Healthy Work Environment Standards or provides evidence‐based methods for integrating spiritual care into clinical settings to support nurse wellness [2]. These gaps limit healthcare leaders’ ability to develop organizational strategies rooted in spiritual principles that address burnout, moral injury, and ongoing workforce shortages. A narrative review is therefore needed to consolidate emerging evidence, clarify core spiritual concepts, and identify leadership approaches that can foster spiritually supportive and sustainable work environments.

### 1.1. Review Questions

1. What is the role of spirituality in facilitating a HWE and nurse wellness?
2. What are the benefits of a spiritually supportive environment to the patient, nurse, and the organization?
3. How can organizations facilitate a spiritually supportive environment?

## 2. Methods

To ensure a comprehensive and nuanced understanding of the development and maintenance of a spiritually HWE, an extensive and systematic search strategy was employed to capture a wide array of perspectives, empirical findings, and expert opinions. The literature search was conducted using the following databases: PubMed, CINAHL, and ProQuest. A combination of keywords and Boolean operators was used to optimize the search for relevant studies. Key search terms included “spirituality in healthcare,” “spiritually supportive environments,” and “AACN healthy work environment.”

### 2.1. Inclusion Criteria

Studies were eligible for inclusion if they met the following criteria: (1) published in peer‐reviewed journals between 2020–2025 (note: a few articles published prior to 2020 were retained due to their relevance and contributions; this range was expanded upon further review); (2) written in English; and (3) focused on healthcare settings. Studies that were originally published in other languages but had reliable English translations were also considered. Studies were excluded if they did not pertain to healthcare contexts.

A narrative review was selected because spirituality in healthcare includes multiple complex and diverse studies that need a comprehensive analysis. The research field contains diverse studies that use different methods to investigate spirituality in healthcare. The complex nature of the research field makes traditional systematic reviews insufficient for uniting findings from different study types. The method of narrative review works best for research topics that need interpretive analysis and flexible evidence integration to answer questions about meaning, context, and application [14]. The narrative review approach proved best for studying spirituality in healthcare because it needed to connect diverse research areas, including nursing practice, organizational behavior, patient results, workforce health, and leadership development.

### 2.2. Literature Selection Process

**FIGURE 1:**
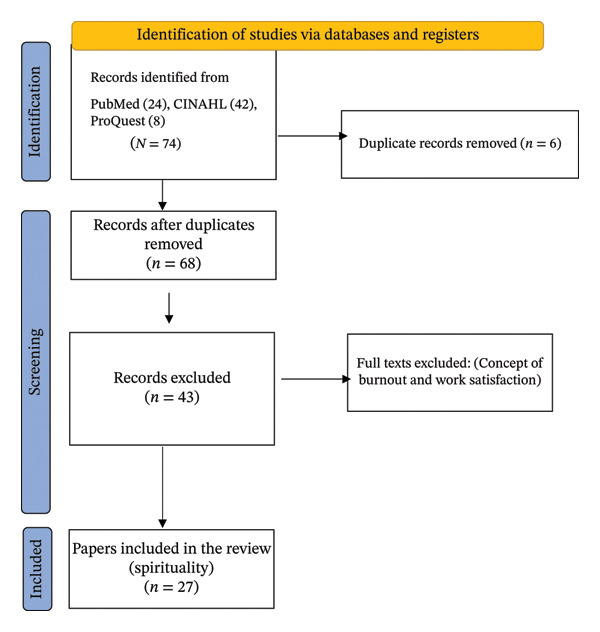
PRISMA flowchart. Note. Adapted from “The PRISMA 2020 statement: An updated guideline for reporting systematic reviews,” by M. J. Page, J. E. McKenzie, P. M. Bossuyt, I. Boutron, T. C. Hoffmann, C. D. Mulrow, L. Shamseer, J. M. Tetzlaff, E. A. Akl, S. E. Brennan, R. Chou, J. Glanville, J. M. Grimshaw, A. Hróbjartsson, M. M. Lalu, T. Li, E. W. Loder, E. Mayo‐Wilson, S. McDonald, Moher, D. 2021, BMJ, 372(71). https://doi.org/10.1136/bmj.n71.

The study selection process involved an initial screening of titles and abstracts conducted independently by multiple reviewers. Microsoft Excel was used to organize and manage references during the screening process. A data extraction template was developed and included the following elements: Author(s), publication year, study design, sample characteristics and setting, instruments or theoretical frameworks, key findings, and implications or recommendations. Full‐text articles were retrieved and reviewed for relevance and alignment with the inclusion criteria. Twenty‐seven articles were included in the final review. Additionally, websites of major professional organizations were examined to gain information on training, research, and practical applications. A Literature Selection Flow Diagram (Figure 1) is added. A literature matrix can be added as a supporting file (available here).

### 2.3. Data Analysis

The researchers evaluated all extracted data by organizing it into categories that matched the three review questions. The research team used a deductive narrative synthesis to identify common elements across studies. Articles were analyzed by comparing their shared elements and distinct points to identify recurring themes about spirituality, nurse well‐being, and HWEs. The authors performed manual coding, organizing their key findings into six categories: patient benefits, nurse benefits, organizational results, leadership effects, obstacles, and support factors. The synthesis was deductive rather than inductive because findings were organized according to three pre‐established review questions, providing a structured framework for categorization. The method enabled the team to merge diverse research approaches into a unified analysis that met the narrative review objectives while preserving methodological clarity throughout the literature review.

## 3. Results Synthesis

Overall, the research findings from this review show that spirituality creates positive work environments by boosting nurse wellness, reducing burnout, building resilience, and delivering better patient outcomes [5, 9]. Research indicates that environments that support spirituality led to better communication, stronger nurse–patient bonds, and safer emotional environments when leaders use transformational methods, when staff receive training, and when organizations create supportive policies and practice reflection [23, 24]. While these studies reveal positive trends, researchers point out that definitions of spirituality remain inconsistent, and organizations provide different levels of support while healthcare staff face ongoing obstacles, including insufficient training, insufficient time, unclear roles, and institutional barriers [25, 26]. The findings indicate persistent gaps, particularly the need for researchers to develop standardized instruments for measuring spiritual support and to establish effective methods for integrating such support within multidisciplinary teams, as well as for examining its implementation across diverse healthcare settings [27, 28]. The review also reveals key evidence gaps, including the need for consistent definitions of spirituality, HWEs, and provider wellness.

The 27 included studies represented a range of research designs, including cross‐sectional surveys (e.g., Refs. [15, 23]), qualitative descriptive studies (e.g., Ref. [25]), scoping reviews (e.g., Refs. [9, 26, 29]), integrative reviews (e.g., Ref. [28]), and framework‐based analyses (e.g., Ref. [30]). Areas of strong agreement across studies included the positive association between spiritual support and reduced burnout, the role of transformational leadership in fostering supportive environments, and the need for training in spiritual care competencies. However, notable differences emerged regarding how spirituality was defined and measured. Some studies used validated instruments such as the Spiritual Well‐Being Scale, while others relied on qualitative self‐report or proxy measures, making direct comparisons difficult. Additionally, while most studies examined spirituality’s positive effects, fewer addressed potential risks or negative consequences, such as spiritual distress arising from moral conflict or the imposition of spiritual values in diverse clinical settings. These patterns suggest that while the evidence base is growing, the field would benefit from greater methodological consistency and more balanced examination of both beneficial and adverse dimensions of spiritual integration in healthcare.

### 3.1. Spirituality and HWEs

Spirituality plays a pivotal role in fostering HWEs and promoting nurse wellness in healthcare settings. Defined as a sense of connection to something greater than oneself, spirituality encompasses personal beliefs, values, and practices that provide meaning and purpose [6]. In nursing, spirituality is not limited to religious practices but extends to fostering resilience, reducing burnout, and enhancing overall well‐being, which are critical components of a HWE as outlined by the AACN [2]. This connection underscores how a spiritually supportive work environment aligns with the AACN’s standards for HWEs, which emphasize skilled communication, genuine collaboration, effective decision‐making, appropriate staffing, meaningful recognition, and authentic leadership [2]. As a result, a HWE will reduce burnout, suicide, mental health issues, and attrition and enhance job satisfaction [21, 31–37]. Spirituality contributes to these standards by fostering a culture of compassion, trust, and mutual respect among healthcare teams. For example, spiritual leadership, which emphasizes values such as altruism and purpose, has been shown to enhance work engagement and organizational commitment among nurses [23]. Such leadership creates an environment where nurses feel valued and supported, reducing turnover intentions and promoting job satisfaction [24].

Furthermore, spiritually supportive environments foster cohesive teamwork and effective communication, which are key elements of HWEs. When nurses feel spiritually supported, they are more likely to engage in collaborative practices and develop trusting relationships with colleagues and patients [38]. This sense of connection not only improves team dynamics but also enhances nurse–patient relationships, enabling nurses to provide holistic, patient‐centered care as advocated by the American Nurses Association [4]. By incorporating spiritual support into the workplace, organizations can foster an environment where nurses flourish, resulting in enhanced job satisfaction and lower burnout rates and emotional exhaustion [39, 40]. Overall, spirituality can be considered a cornerstone of a HWE and nurse wellness in healthcare settings.

### 3.2. Spirituality and Nursing (Healthcare Provider Wellness)

By providing spiritual support, an organization enhances the well‐being of patients, empowers healthcare workers, and strengthens itself overall. Patients will receive cohesive treatment, the team will be harmonious, and organizations will gain a reputation. These are described below.

#### 3.2.1. Benefits to the Patient

1. Improved outcomes—improved quality of life, especially in those with serious illness [5].
2. Better mental health—receive holistic care; spiritual support helps reduce anxiety, depression, and stress, contributing to better mental health [27, 41].
3. Better pain management, coping, and faster recovery resulting in financial gain for the patient [5, 7].
4. Patient satisfaction with hospital stays and the quality of care as indicated by Press Ganey scores [5, 28].

#### 3.2.2. Benefits to the Nurse/HealthCare Provider

1. Addressing burnout, fostering resilience, and supporting nurses emotionally and in conflict resolution contribute to stronger nurse–patient relationships based on trust, empowering nurses to deliver holistic care [9, 15, 20, 41–43].
2. Increase job satisfaction when the nurse provides meaningful support to the patients and establishes a positive relationship with them. The teamwork will be cohesive, teamwork together or teamwork with the patient [27, 44, 45].
3. Professional growth in the nurses where they will develop skills to deal with people of different cultures and faiths [5, 27].
4. Nurses’ well‐being improves when they engage in reflective practice and experience a strong sense of purpose. Such well‐being is cultivated within a supportive environment that encourages ethical conduct and spiritual support [5, 8, 9].

#### 3.2.3. Benefits to the Organization

1. It enhances the patient experience, patient satisfaction, and improved patient outcome [5].
2. Workforce well‐being is supported by providing spiritual care, which reduces burnout and boosts job satisfaction [5, 11, 27, 46].
3. Organizational reputation will increase, there will be a positive image in the community so that they can recruit and retain staff. The organization will be known for ethical practice and will support collaborative partnerships, strong communication, and research and innovation [27].

### 3.3. Challenges, Barriers, and Facilitators to Implementation

Although providing spiritual care offers benefits for nurses, patients, and families, and healthcare organizations, it is also accompanied by notable challenges and barriers. A list of benefits, challenges, and barriers is given in Table 1. Daaleman [30] reviewed three studies in the clinical arena using the quality framework of structure, process, and outcome described by Donabedian [47]. Daaleman identified barriers to spiritual care such as lack of time, social, cultural, demographic differences, and institutional obstacles, such as lack of privacy and continuity. Similarly, other studies have reported additional challenges, such as lack of preparedness, lack of time, fear of offending patients, uncertainty about how to provide spiritual care, role ambiguity, poor work environment, limited institutional support, lack of privacy, and belief that spirituality is a private matter, as barriers to providing spiritual care [25, 26]. Conversely, facilitators include time, training, coherent communication, and transparency about personal experience [28, 30, 48]. Addressing these is important for the interdisciplinary implementation of spiritual caregiving, where the staff are equipped and patients are cared for well. Wisuda et al. [49] reported a significant correlation between nurses’ spiritual care practices and patients’ spiritual fulfillment. However, all nurses are expected to give spiritual care to patients of all faiths.

**TABLE 1:** Cultivating spiritually supportive work environments in healthcare organizations.

Steps	Barriers	Challenges	Benefits
1. Integrate Spiritual Care into Clinical Practice	Lack of training in spiritual care [5]	Differentiating spirituality from religion, time constraints in clinical settings [5]	Enhances holistic care; improves patient satisfaction and trust [5]
2. Promote Spiritually Competent Practice	Ambiguity in defining spirituality [7]	Ensuring cultural sensitivity and inclusivity [7]	Supports meaning‐making and resilience in patients and staff [7]
3. Train Staff Using Structured Curricula (e.g., ISPEC)^∗^	Limited institutional support or funding [5]	Resistance to change; lack of awareness [5]	Builds confidence and competence in addressing spiritual needs [5]
4. Foster Compassionate Leadership and I‐Thou Relationships	Hierarchical structures that discourage vulnerability [7]	Balancing technical and relational care [7]	Encourages empathy, trust, and psychological safety [7]
5. Create Inclusive Policies and Reflective Spaces	Institutional rigidity, lack of policy [27]	Balancing diverse spiritual expressions [27]	Promotes diversity and inclusion, psychological safety, and employee well‐being [27]
6. Measure and Evaluate Spiritual Care Outcomes	Lack of standardized metrics [27]	Difficulty in quantifying spiritual impact [27]	Demonstrates value; informs continuous improvement [27]

^∗^Interprofessional Spiritual Care Education Curriculum developed by the George Washington Institute for Spirituality and Health.

Beyond barriers to implementation, it is also important to acknowledge that spirituality in healthcare may carry potential negative consequences if not approached thoughtfully. For example, when spiritual care lacks cultural sensitivity, patients or staff from minority faith traditions may feel marginalized or pressured to conform to dominant religious perspectives [26]. Spiritual distress can arise when clinicians encounter moral conflicts between their personal spiritual beliefs and the care they are expected to provide, potentially compounding rather than alleviating emotional strain [29]. Additionally, an overemphasis on spirituality as a coping mechanism may inadvertently shift responsibility for well‐being from the organization to the individual, thereby masking systemic issues such as inadequate staffing, excessive workloads, or toxic work cultures [22]. Organizations that promote spiritual wellness programs without simultaneously addressing structural determinants of distress risk fostering a culture of spiritual bypassing, wherein deeper organizational dysfunction remains unexamined. These considerations underscore the importance of integrating spiritual support within a broader, system‐level approach to workforce well‐being.

### 3.4. How Can Organizations Facilitate a Spiritually Supportive Environment?

Cultivating spiritually supportive work environments in healthcare is increasingly recognized as essential for enhancing staff well‐being, improving patient care, and fostering organizational resilience. Research has identified several evidence‐based strategies to achieve this goal, with transformational leadership playing a critical role in their success.

#### 3.4.1. Transformational Leadership: A Catalyst for a Spiritually HWE

Transformational leadership plays a crucial role in creating healthcare environments that support nurses’ professional well‐being and their spiritual and emotional needs. Such leaders foster trust, meaning, and resilience by inspiring a shared vision, recognizing individual contributions, and cultivating authentic relationships [50–52]. By modeling ethical behavior and encouraging holistic growth, transformational leaders help align organizational practices with nurses’ intrinsic desire for purpose, dignity, and connection in their work.

Research demonstrates that transformational leadership significantly improves job satisfaction, strengthens organizational commitment, and promotes personal mastery among nurses and other healthcare professionals [18, 53]. Most importantly, transformational leaders bridge professional practice with spiritual well‐being by fostering environments where values such as compassion, fairness, and hope are respected and nurtured [53–55]. When nurses perceive their leaders as supportive of their spiritual and religious needs, they are more likely to experience less burnout, higher engagement, greater emotional resilience, and find a stronger sense of meaning and purpose in work [23, 24, 56–59]. In advancing healthy, spiritually supportive workplaces, transformational leadership becomes not just a managerial style but serves as a vital ministry of healing, dignity, and hope within healthcare systems. Notably, postpandemic research affirms that transformational leadership remains a powerful predictor of HWEs, underscoring the value of leaders who honor the full humanity of their teams, including their emotional, spiritual, and relational dimensions [60]. To this end, nurse leaders and managers must be equipped to identify signs of moral injury and foster environments that prioritize ethical decision‐making, staff well‐being, and emotional safety, and develop resiliency to equip nurses to provide quality care [11, 12, 22, 28, 61]. Organizations should integrate spiritual leadership principles to foster a value‐driven, resilient, and ethical workplace culture, and leaders must be trained to nurture meaning and purpose in their employees [19, 45, 62].

#### 3.4.2. Spiritual Care Integration in the Practice of the Multidisciplinary Team

A multidisciplinary team must be capable of integrating spirituality in all aspects of patient care and teamwork. Evidence‐based strategies to foster a spiritually supportive workplace include the following:

1. Assess spirituality during the routine initial interview with the patient.
2. Educate nurses and all appropriate staff on how to complete a spiritual assessment [16, 29].
3. Equip all healthcare providers for spiritual assessment as part of their educational programs so that they can integrate it when they enter practice [28].
4. Support impact sharing circles, employee‐led spiritual initiatives, and peer mentoring [63].
5. Provide orientation programs to include a hands‐on training on spiritual assessment and integration [17, 28, 58, 64, 65].

#### 3.4.3. Facilitation of Structure and Resource Support for Spiritual Care

Organizations can provide a supportive environment by providing physical space for spiritual practice or reflection and respecting different types of faiths [63]. In addition, spiritual care teams can be hired, and organizations can collaborate with community organizations so that the standing of the organization in the community will be established or furthered. By providing space and resources, organizations can encourage practices that support physical, mental, and spiritual health, such as exercise, meditation, and adequate rest [59, 66]. Thereby, organizations can help staff maintain balance and resilience.

Spiritual support for the staff and patients must become embedded in the organizational culture [59]. Establishing this supportive culture can help organizations remain accreditation‐ready at all times. For example, Hospice New Zealand has developed a spirituality professional development program [41]. Similarly, Daaleman [30] applied a health services framework to explore spiritual care providers, patients, and family members who experienced death in a long‐term care facility. Notably, spiritual support often includes practices such as prayer, and several studies explored the effect of prayer on the health and recovery of patients [67]. Burgos [68] reported that digital competencies can enhance the nurses’ ability to provide spiritually sensitive care, and advocates for enhancing infrastructure to provide holistic care, including spiritual care.

### 3.5. Implications of Creating and Sustaining a Spiritually Supportive Healthcare Environment

Based on the collective evidence, creating and sustaining a spiritually supportive healthcare environment carries profound implications for both workforce vitality and patient care, fostering a culture where meaning, compassion, and holistic well‐being are integral to organizational success. Fostering a spiritually supportive environment requires buy‐in from the leadership in both educational and service organizations. Additionally, it must be integrated into education so that the students who graduate enter the workforce with a positive attitude toward the integration of spirituality in their day‐to‐day services.

### 3.6. Implications for Leadership

The integration of spirituality into healthcare work environments has significant implications for nursing leadership. Transformational leaders inspire, motivate, and support their teams by fostering a shared vision that integrates spirituality into the core culture of the healthcare environment. These leaders are well‐positioned to cultivate spiritual supportive environments that align with the AACN’s Health Work Environment standards (2016). By exemplifying core values such as empathy, authenticity, and purpose, transformational nurse leaders can foster a culture where meaning, connection, and respect are embedded in daily nursing practice. When transformational leadership is coupled with a focus on spiritual well‐being, nurses are more likely to feel supported and connected to a greater sense of purpose, which can lead to improved collaboration, reduced turnover, and higher job satisfaction [11, 69]. Moreover, transformational leaders can operationalize spirituality in the workplace by encouraging reflective practice, facilitating open dialog about values and purpose, and recognizing the holistic contributions of nursing. Ultimately, investing in leadership development that integrates spiritual awareness with transformational leadership principles is essential to creating HWEs where both staff and patients can benefit. Similar to the AACN [2] HWE, the Registered Nurses’ Association of Ontario [70] developed guidelines for managers about a HWE, which covers cognitive, psychosocial, and cultural factors. Transformational leaders are pivotal in fostering a HWE that fosters increased staff emotional health and decreased staff burnout, thereby enhancing retention.

### 3.7. Implications for Education

Integrating spirituality into nursing education holds profound implications for preparing future nurses to provide holistic, patient‐centered, and compassionate care. Academic nursing programs should systematically incorporate spiritual care concepts throughout the curriculum, ensuring that the development of spiritual competence is addressed alongside clinical knowledge and technical proficiency. This integration involves equipping students with the skills to assess and respond to patients’ spiritual needs, fostering reflective practice, and cultivating cultural and spiritual sensitivity. Dewi et al. [71] found that spiritual care competencies taught in nursing schools have improved the students’ ability to provide holistic, patient‐centered care to people of different religious and spiritual contexts. Furthermore, faculty play a pivotal role in this process by modeling spiritually attuned care and cultivating educational environments that support the personal and professional formation of students. Pedagogical strategies such as simulation‐based learning, case studies, and clinical experiences should intentionally include spiritually relevant scenarios to enhance students’ empathy, confidence, and clinical judgment. In doing so, nursing education can contribute to the formation of practitioners who are not only clinically competent but also spiritually responsive and ethically grounded [28]. Selected organizational and educational resources that can support the development of spiritually supportive structures and programs are summarized in Table 2.

**TABLE 2:** Selected resources for spiritual care in healthcare.

Organization	About	Description	Weblink
American Nurses Association	Healthy Nurse, Healthy Nation	A mission to inspire a healthy world through the power of nursing with Nurse Burnout Prevention Programs and other free training.	https://www.healthynursehealthynation.org/
Duke University	Center for Spirituality, Theology and Health	Offering monthly webinars, publications, and workshops on spirituality in health	https://spiritualityandhealth.duke.edu
EPICC Network	EPICC Network is for those interested in nursing/midwifery spiritual care education and practice	Enhancing Nurses’ and Midwives’ Competence in Providing Spiritual Care through Innovative Education and Compassionate Care	https://epicc-network.org/
George Washington University	ISPEC Curriculum	Interprofessional Spiritual Care Education Curriculum for clinicians	https://smhs.gwu.edu/gwish/education/ispec
Harvard University	Human Flourishing Program—Forgiveness Project	Examining the role of forgiveness in flourishing and healthcare	https://hfh.fas.harvard.edu/forgiveness
INSS—International Network for the Study of Spirituality	International Network for the Study of Spirituality	Study of spirituality to life through research, scholarship, education and practice	https://spiritualitystudiesnetwork.org/
Johns Hopkins University	Forgiveness and Spirituality in Wellness	Exploring how forgiveness impacts physical, mental, and spiritual health.	https://hopkinsmedicine.org
Liberty University	Global Center for Human Flourishing	A collaborative project between researchers at Harvard University and Baylor University, partnering with Gallup to collect extensive data on human flourishing across diverse countries.	https://www.liberty.edu/academic-affairs/global-center-for-human-flourishing/
Mercy College	Center for Human Flourishing	Providing knowledge through research publications, sponsored educational activities, courses, seminars, and conferences of higher learning, particularly in the fields of health.	https://www.mchs.edu/experience/center-for-human-flourishing/
RNAO (Canada)	Healthy Work Environment Guidelines	Offering leadership and staff tools for spiritually healthy workplaces	https://rnao.ca/sites/rnao-ca/files/HWE_Reference_Guide.pdf

There are models of training available as found in the Interprofessional Spiritual Care Education Curriculum (ISPEC) developed by the George Washington Institute for Spirituality and Health to equip clinicians [5]. Such training will help clinicians recognize spiritual distress in clients, engage in meaningful conversations, and support their spiritual well‐being. Spirituality as a topic of interest is evident when universities such as Harvard host the forgiveness movement [72] and Duke hosts the Center for Spirituality [73]. In addition, Duke University hosts monthly free webinars, newsletters, and regular conferences and workshops. These resources will be useful to equip emerging healthcare professionals (Table 2). Other major organizations, such as Johns Hopkins University [74], foster spirituality and forgiveness for enhancing the health of their stakeholders, which includes their employees, patients, and communities.

### 3.8. Implications for Practice

Nurses who are adequately prepared and supported to assess and respond to patients’ spiritual needs are better equipped to foster meaningful therapeutic relationships, enhance emotional well‐being, and contribute to more comprehensive and person‐centered healing processes. Spiritual care practices can alleviate spiritual and existential distress, factors often associated with anxiety, depression, and diminished coping capacity. In addition, spiritually informed nursing practice promotes ethical sensitivity, cultural competence, and compassionate communication, all of which are essential in delivering high‐quality care in diverse clinical environments. By embedding spiritual awareness into everyday practice, nursing moves beyond its technical dimensions to embrace a more humanistic approach. This approach strengthens the nurse–patient relationship and reinforces the moral and relational foundations of professional nursing.

The case‐based and reflective learning offered in the ISPEC fosters collaboration with spiritual care specialists for more complex needs [5]. Organizations can learn from the RNAO [70] report that describes competencies for nurse leaders and sample behaviors and strategies for creating an empowering work environment where nurses can flourish as a team. Shamsi et al. [48] support ongoing professional development for nurses, and Gad et al. [75] advocate for the initiation of spiritual care irrespective of the spiritual/religious background of the patients. The spiritual care model developed by Ghorbani et al. [76] provides a structured guide for nurses to integrate spiritual care into clinical practice.

### 3.9. Implications for Research

The growing body of research linking transformational leadership with enhanced job satisfaction, organizational commitment, and spiritual well‐being underscores the need for continued empirical investigation into how spiritual care interventions influence patient outcomes, staff well‐being, and organizational culture. This includes developing and validating tools to assess spiritual needs, evaluating the effectiveness of spiritually integrated care models, and identifying best practices for diverse populations. Furthermore, nursing research must address gaps in evidence by conducting studies on the role of spirituality in reducing burnout, enhancing resilience, and improving communication and empathy in clinical settings. Furthermore, interdisciplinary research is needed to evaluate the effectiveness of evidence‐based strategies for integrating spiritual care into the practice of multidisciplinary teams. This includes investigating the outcomes of spiritual assessments, training, and the organizational benefits of providing dedicated spiritual care resources and reflective spaces. Comparative studies across institutions that implement varying degrees of spiritual care integrations would be valuable in identifying best practices and scalable interventions. Studies that explore the effect of prayer on health outcomes can be undertaken. The predictive value of spiritual integration on the reduction of burnout of staff in different specialty areas can be explored [77, 78]. In addition, tools must be developed to measure different dimensions of spiritually integrated leadership, service, and outcome in nurses [49, 62]. Ultimately, continued research is essential to inform policy, education, and practice innovations that promote spirituality as a core component of workforce well‐being and high‐quality, patient‐centered care. Specifically, future research should pursue the following priorities: (a) developing and validating culturally inclusive instruments for measuring spirituality, spiritual well‐being, and spiritual care competence across diverse healthcare populations; (b) conducting longitudinal and interventional studies to establish causal relationships between spiritual support programs and workforce outcomes such as burnout reduction, retention, and job satisfaction; (c) investigating the impact of spiritual support on multicultural and interprofessional teams, particularly in settings with religiously and culturally diverse staff and patient populations; (d) examining potential adverse effects of spiritual integration, including spiritual distress, cultural imposition, and organizational spiritual bypassing; (e) applying implementation science frameworks to evaluate the feasibility, adoption, and sustainability of spiritual care interventions in diverse clinical settings; and (f) establishing clear evaluation metrics that organizations can use to assess the effectiveness of spiritually supportive workplace initiatives over time.

### 3.10. Limitations

Several limitations should be considered when interpreting the findings of this narrative review. First, as a narrative rather than systematic review, the literature selection process did not follow a standardized protocol such as PRISMA, and the inclusion of studies was guided by the authors’ judgment regarding relevance and fit with the review questions. This approach, while appropriate for synthesizing a broad and conceptually diverse body of literature, introduces the possibility of selection bias. Second, the majority of included studies were cross‐sectional in design, which limits the ability to draw causal inferences about the relationship between spirituality, nurse well‐being, and organizational outcomes. Third, definitions of spirituality varied considerably across the reviewed studies, ranging from religiosity‐focused constructs to broader existential and meaning‐making frameworks. This inconsistency makes it difficult to compare findings directly or to establish a standardized operational definition for clinical application. Fourth, most studies were conducted in Western healthcare contexts, which may limit the generalizability of findings to non‐Western or culturally distinct settings where spirituality may be understood and expressed differently. Cultural biases inherent in existing instruments and frameworks may not adequately capture the spiritual experiences of diverse populations. Fifth, the review identified few studies that examined the potential negative consequences of spiritual integration, such as spiritual coercion, cultural insensitivity, or the displacement of systemic interventions with individual‐level spiritual programs. This gap represents an important area for future inquiry. Finally, the practical challenges of implementing spiritual support in high‐acuity, fast‐paced clinical environments were acknowledged but not extensively examined in the reviewed literature, suggesting a need for implementation science approaches that account for real‐world clinical constraints.

## 4. Conclusion

This narrative review examined the role of spirituality in facilitating HWEs and nurse wellness, the benefits of spiritually supportive environments for patients, nurses, and organizations, and strategies through which organizations can cultivate such environments. Regarding the first review question, the evidence suggests that spirituality contributes to HWEs by fostering resilience, reducing emotional exhaustion, and supporting a sense of meaning and purpose among nurses [9, 23]. These findings align with the AACN Healthy Work Environment Standards, particularly in the domains of authentic leadership, meaningful recognition, and skilled communication [2]. Concerning the second question, the reviewed literature indicates that spiritually supportive environments may improve patient outcomes including satisfaction and quality of life, enhance nurse well‐being and job satisfaction, and contribute to organizational benefits such as improved retention and a strengthened reputation [5, 24, 27]. In addressing the third question, findings point to transformational leadership as a key catalyst for creating spiritually supportive workplaces, supported by strategies such as spiritual care training, multidisciplinary integration, provision of reflective spaces, and organizational policies that embed spiritual support into institutional culture [28, 58]. However, these conclusions should be interpreted with caution, given the heterogeneity in how spirituality was defined and measured across studies, and the predominance of cross‐sectional designs that limit causal inference. Future research should prioritize the development of standardized measurement tools, conduct longitudinal and interventional studies, and examine both the benefits and potential risks of spiritual integration in diverse healthcare settings. These efforts are essential to inform evidence‐based policy, education, and practice innovations that position spiritual support as a meaningful component of workforce well‐being and high‐quality, patient‐centered care.

## Author Contributions

Conceptualization: Rachel A. Joseph and Ashley Hudson Tharpe.

Data collection: Rachel A. Joseph, Holly Ortman, and J. Michelle Crager.

Writing/revising/editing: Rachel A. Joseph, Holly Ortman, J. Michelle Crager, and Ashley Hudson Tharpe.

## Funding

This research received no specific grant from any funding agency in the public, commercial, or not‐for‐profit sectors.

## Conflicts of Interest

The authors declare no conflicts of interest.

## Supporting Information

Literature matrix spiritually supportive work environments.

This literature matrix contains the relevant articles reviewed on this topic.

## Supporting information


**Supporting Information** Additional supporting information can be found online in the Supporting Information section.

## Data Availability

The data supporting this review are from previously reported studies and datasets, which have been cited. The processed data are available from the corresponding author upon request. Data sharing does not apply to this manuscript as no datasets were generated or analyzed during the current study.
